# Recurrent acute pancreatitis during a ketogenic diet—a case report and literature review

**DOI:** 10.1186/s12245-021-00374-5

**Published:** 2021-09-15

**Authors:** Joseph Choi, Tayler L. Young, Lucas B. Chartier

**Affiliations:** 1 Emergency Department, University Health Network, 200 Elizabeth Street, RFE – Ground Floor, 480, Toronto, Ontario M5G 2C4 Canada; 2 Division of Emergency Medicine, Department of Medicine, University of Toronto, C. David Naylor Building, 6 Queen’s Park Crescent West, Third Floor, Toronto, Ontario M5S 3H2 Canada; 3 Faculty of Medicine, University of Ottawa, 451 Smyth Road, Ottawa, Ontario K1H 8M5 Canada

**Keywords:** Pancreatitis, Ketogenic diet, Low carbohydrate diet, Gastroenterology, Emergency department

## Abstract

**Background:**

The ketogenic (“keto”) diet has been gaining more attention lately in the medical literature and the lay media as a potentially effective method for weight control and management of type 2 diabetes. Though rare, there have been case reports of serious side effects. Here, we present a peculiar case of pancreatitis presumably associated with the ketogenic diet.

**Case presentation:**

A 35-year-old man on a calorie-restricted ketogenic diet presented to the emergency department with weekly abdominal pain on Monday mornings, each time after dietary indiscretions (“cheat days”) on the weekend. It was found that he had a clinical presentation consistent with acute pancreatitis with no associated alcohol use, hypertriglyceridemia, pancreatic obstruction, or other anatomic abnormalities. The patient’s symptoms resolved with conservative management and progressive reintroduction of a standard diet.

**Conclusion:**

This case indicates that the ketogenic diet could lower the threshold for acute pancreatitis, and that an episodic stressor may trigger an acute attack in the absence of traditional risk factors.

## Background

Low-carbohydrate diets for weight management have been described since the 1960s with the popularization of various diets such as the Atkins, Paleo, and ketogenic diets, and their popularity have been growing since [1]. Given the increasing prevalence of obesity [2] and diabetes [3], there has been an increasing interest in the role of carbohydrate consumption in the pathogenesis of these two conditions. Indeed, there is literature that suggests a low-carbohydrate diet is effective in managing these two conditions [4–8], but larger studies are required to determine its long-term effects and generalizability [9].

While complications associated with the ketogenic diet are relatively rare [10], there have been reports of serious adverse events, including cardiac effects (such as dysrhythmias and sudden cardiac death) [11, 12], hepatitis, and ketoacidosis [13]. To date, there is only one published case report of acute pancreatitis associated with the use of a ketogenic diet for weight loss [14]. That case report presents an adult patient who had a severely elevated triglyceride level at the time of admission and pre-existing hypertriglyceridemia being controlled by medications.

The case we describe below is the first to describe a case of acute pancreatitis while on the ketogenic diet in the absence of severe hypertriglyceridemia.

## Case presentation

A 35-year-old obese man presented to the emergency department (ED) with severe central abdominal pain with mild radiation to his back. He also had nausea and one episode of non-bilious, non-bloody vomiting. He denied fever or any change in his bowel habits. His review of systems was otherwise unremarkable. He denied any recent travel history, had not been exposed to any chemicals or exotic animals, and was in his usual state of health otherwise. He did not describe any infectious symptoms leading up to the presentation to the ED.

His past medical history was significant for hypertension, gout, type 2 diabetes mellitus, and dyslipidemia (elevated triglycerides, elevated total cholesterol, and high total cholesterol/HDL ratio). He was not on any medications, and these conditions were all managed through diet and lifestyle modifications under the supervision of his primary care provider (PCP). He had no surgical history. Although he reported being a heavy drinker in his early 20s, he had cut back significantly for many years and only had three drinks in the month prior to presentation. He had a 15 pack-year smoking history, but he transitioned to smokeless tobacco products and nicotine replacement products over the preceding 3 years. He occasionally ingested marijuana recreationally but did not smoke it. He denied any other substance or supplement use.

On further questioning, the patient reported having been on a 2000-calorie-a-day ketogenic diet for the past 3 weeks, with “cheat days” on the weekends when he ate whatever he wanted. He did not track the exact content of his diet on these cheat days, but he consumed far more carbohydrates relative to his diet days. After these cheat days, he would resume his ketogenic diet the following Mondays. The diet was designed by himself with advice from Internet sources, and he had a discussion with his PCP who had given him his approval. The patient reported having experienced identical but less severe symptoms on Mondays since starting his diet (as it was on the day of this presentation). The first Monday he experienced these symptoms, he had presented to a walk-in clinic but no definitive diagnosis was made. The second episode of pain occurred the following Monday, which was more severe and sustained. This led him to present to the ED.

The patient’s initial vital signs included a blood pressure of 155/101 mmHg, a heart rate of 77, a respiratory rate of 18, an oxygen saturation of 95% on room air, and he was afebrile. They remained similar and stable throughout the patient’s ED course.

On examination, the patient appeared well but in pain, rated as 10/10. He was not jaundiced, and his mucous membranes were moist. His cardiorespiratory examination was unremarkable. The patient’s abdomen was non-tympanic with normal bowel sounds and no skin discoloration. The patient was exquisitely tender in the epigastrium, but it was otherwise soft without peritoneal signs. He had neither right upper quadrant tenderness nor Murphy’s sign. Point-of-care (i.e., bedside) ultrasound showed no abdominal free fluid, no gallstones, and no other sonographic signs of cholecystitis.

The primary differential diagnosis considerations included hepatobiliary causes (such as biliary colic, choledocholithiasis, and acute cholecystitis), pancreatic causes (such as acute pancreatitis), and gastric causes (such as gastritis, gastric and duodenal ulcers) given the upper abdominal tenderness and vomiting. An atypical presentation of acute coronary syndrome was also considered, but was less likely given the history and physical examination findings. Similarly, thoracic and pulmonary diagnoses were entertained, but did not seem likely given the details of the case.

The patient’s point-of-care glucose testing was 9.3 mmol/L. His electrocardiogram showed normal sinus rhythm. The patient’s bloodwork on presentation to the ED is shown in Table 1. The only significant abnormalities were an extremely high lipase (2283 U/L), an elevated glucose (9.3 mmol/L), and an elevated white blood cell count (15.4 × 10^9 cells/L).

**Table 1:** Patient’s laboratory profile on admission to the ED. Abnormal lab values are in bold

Blood test	Value	Normal range
Complete blood count		
Hemoglobin	151 g/L	140-180 g/L
White blood cells	**15.4** × **10^9/L**	4-11 × 10^9/L
Platelets	223 × 10^9/L	150-400 × 10^9/L
Hematocrit	0.43	0.42-0.54
Na	139 mmol/L	135-145 mmol/L
K	4.7 mmol/L	3.2-5.0 mmol/L
Cl	103 mmol/L	100-110 mmol/L
HCO3	23 mmol/L	23-29 mmol/L
Cr	83 μmol/L	64-110 μmol/L
eGFR	> 104 mL/min/m^2^	> 60 mL/min/m^2^
Glucose	**9.3 mmol/L**	3.8-7.0 mmol/L
Ca	2.49 mmol/L	2.20-2.62 mmol/L
Mg	0.70 mmol/L	0.70-1.10 mmol/L
PO4	0.98 mmol/L	0.80-1.40 mmol/L
Aspartate aminotransferase (AST)	34 U/L	5-34 U/L
Alanine aminotransferase (ALT)	**43 U/L**	7-40 U/L
Alkaline phosphatase (ALP)	70 U/L	40-150 U/L
Bilirubin	11 μmol/L	< 22 μmol/L
Lipase	**2283 U/L**	< 60 U/L
Troponin	2 ng/L	< 26 ng/L
Lactate	0.8 mmol/L	< 2 mmol/L
IgG subclass analysis		
IgG1	5.85	3.82-9.29
IgG2	**2.25**	2.42-7.00
IgG3	0.48	0.22-1.76
IgG4	0.076	0.039-0.864

A comprehensive ultrasound of the abdomen showed severe fatty infiltration of the liver, a normal gallbladder with no gallstones, non-dilated bile ducts, and patent portal and hepatic veins. The pancreas was incompletely visualized due to bowel gas and body habitus but it was described as ill-defined and heterogeneous, but without peripancreatic fluid or other ductal abnormality.

Given the patient’s characteristic signs and symptoms and a significantly elevated lipase, the diagnosis of acute pancreatitis was confirmed, and the patient was admitted to the general surgery service.

On the ward, the patient’s pain was controlled with opioids, and he received anti-emetics and intravenous fluids. Initially set at nil per os, his diet was slowly advanced until he was able to tolerate food without pain, and he was discharged home 2 days later. The patient’s lipase level was never measured again, but all other previously performed blood results were repeated and returned to the normal range within 24 h of admission.

Table 2 compares his lipid and metabolic profile from 3 weeks prior to the hospital admission (and prior to starting the ketogenic diet) to the values obtained on his presentation to ED. It is interesting to note that over the 3 weeks of the ketogenic diet, his metabolic profile generally improved despite the relative increase of calories derived from fat.

**Table 2:** Metabolic profile performed immediately prior to the start of the ketogenic diet compared with the day of ED visit

Test	Normal range	3 weeks prior (before ketogenic diet)	Day of ED presentation
Hemoglobin A1c	< 6%	7%	6.2%
Cholesterol	Variable	5.88 mmol/L	3.94 mmol/L
Triglyceride	< 2.2 mmol/L	8.53 mmol/L	2.51 mmol/L
HDL-cholesterol	Variable	0.65 mmol/L	0.60 mmol/L
LDL-cholesterol	Variable	N/A^a^	2.19 mmol/L
Non-HDL cholesterol	Variable	5.23 mmol/L	3.3 mmol/L
Total cholesterol/HDL ratio	Variable	9.0	6.6

^a^Invalid result when triglycerides are too high

The patient continued to do well after his discharge from the hospital. He abandoned a strict ketogenic diet and reintroduced carbohydrates into his diet, while increasing his fruits and vegetable intake. He had not had any further episodes of acute pancreatitis 6 months after his index admission.

## Discussion and conclusion

The average American diet contains 250-350 g of carbohydrates per day, much of which is comprised of refined starches and added sugars [15]. Ketogenic diets typically restrict carbohydrate intake to less than 50 g/day [16]. This reduction in carbohydrate intake leads to drops in insulin secretion, which in turn decreases lipogenesis and fat deposition. In this setting, the body adapts by activating lipolysis and transitions from glucose to fat as its primary energy source. Metabolism of fatty acids produces acetoacetate and beta-hydroxybutyrate, collectively known as ketone bodies, and the resulting state is referred as ketosis [16].

Acute pancreatitis typically involves inappropriate activation of pancreatic zymogens, leading to acinar cell damage, leakage of these enzymes into the tissues of the pancreas, and pancreatic autodigestion [17]. There are established risk factors and causes for pancreatitis, such as gallstones, alcohol use, and hypertriglyceridemia, that are well described offenders [18].

On literature review, six studies outlining seven cases were found in which acute pancreatitis developed in association with ketogenic diet. Of the 68 papers identified and reviewed, no case was found to describe cases of pancreatitis with normal or near-normal triglyceride levels. The characteristics of these studies are outlined in Table 3. The literature search was conducted with Medline. The following Boolean approach was found to yield the most relevant and comprehensive results: “pancreatitis” AND “ketogenic.” All six studies included surmised that hyperlipidemia secondary to ketogenic diet contributed to the onset of acute pancreatitis. In most cases, triglyceride levels at the time of pancreatitis onset were not disclosed.

**Table 3:** Studies examining the association of pancreatitis and the ketogenic diet

Study	Study type	Number of patients in study	Number of cases of acute pancreatitis	Age, year	Indication for ketogenic diet	Triglycerides at time of diagnosis of acute pancreatitis	Ketogenic diet duration	Outcome of patients with acute pancreatitis
Stewart et al. [19]	Case report	1	1	9	Glucose transport protein syndrome	N/A	8.5 years	Death
Mackay et al. [20]	Retrospective chart review	26	1	Median age in study was 6.1	Refractory epilepsy	N/A	Median duration of treatment in study was 9 months	Early cessation of ketogenic diet, treatment details unknown
Mori et al. [21]	Case report	1	1	5.7	Refractory epilepsy	N/A	5.25 years	Death
Lyczkowski et al. [22]	Retrospective study	71	2	Mean age in study was 6.52	Refractory epilepsy	N/A	4 months	Early cessation of ketogenic diet, treatment details unknown
Sofou et al. [23]	Longitudinal cohort study	19	1	4.2	Pyruvate dehydrogenase complex deficiency	N/A	15 months	Early cessation of ketogenic diet, treatment details unknown
Buse et al. [14]	Case report	1	1	42	Weight loss	> 25.86 mmol/L (normal < 2.2 mmol/L)	Several days	13-day hospital admission with discharge in good condition and normalized triglycerides

Our case report describes the first case report of a patient developing acute pancreatitis in association with the ketogenic diet with near-normal triglyceride levels at the time of diagnosis. The majority of cases in the literature describe pancreatitis associated with the ketogenic diet in children with metabolic syndromes or refractory epilepsy. A particularly interesting feature of this patient’s case is that the recurrent episodes of presumed pancreatitis seemed to be triggered by relatively higher carbohydrate intake and higher caloric intake during his cheat days, as opposed to the sustained higher fat intake during his diet maintenance days. When he would go back to his carbohydrate-restricted, high-fat intake days, the symptoms seemed to resolve on their own over the course of the week. Unfortunately, the patient did not keep a diary to track his exact food intake over these cheat days, and simply stated that he ate “everything bad” without adhering to any limits.

The patient’s triglyceride levels (along with glycated hemoglobin and cholesterol levels) had improved significantly on the day of his presentation to the ED compared to his fasting blood work done by his PCP prior to starting the diet. If the increased proportional fat intake of the ketogenic diet itself were to blame for his recurring bouts, the patient would have had a history of pancreatitis prior to the start of the diet when his fasting triglyceride levels were even higher.

As the popularity of low-carbohydrate diets increase, healthcare providers must be vigilant of potential complications presenting to the emergency department. This case emphasizes lifestyle and dietary factors that contribute to acute disease states and presentations and may be missed by providers during their history taking. While this case did not have any of the risk factors or triggers that are classically associated with acute pancreatitis, we hypothesize that the cyclic and significant fluctuations in dietary composition (and therefore large swings in pancreatic secretory activity) may have contributed to the development of acute pancreatitis in this patient.

## Data Availability

All relevant raw data (lab values, imaging findings) has been included in this manuscript.

## Abbreviations

ED: Emergency department
PCP: Primary care provider
WBC: White blood cells
AST: Aspartate aminotransferase
ALT: Alanine aminotransferase
ALP: Alkaline phosphatase
